# Thyroid Hormone and Mitochondrial Dysfunction: Therapeutic Implications for Metabolic Dysfunction-Associated Steatotic Liver Disease (MASLD)

**DOI:** 10.3390/cells12242806

**Published:** 2023-12-09

**Authors:** Raghu Ramanathan, Sohum A. Patwa, Ahmad Hassan Ali, Jamal A. Ibdah

**Affiliations:** 1 Division of Gastroenterology and Hepatology, University of Missouri, Columbia, MO 65212, USA; raghu.ramanathan@health.missouri.edu (R.R.); aliah@health.missouri.edu (A.H.A.); 2 Harry S. Truman Memorial Veterans Medical Center, University of Missouri, Columbia, MO 65212, USA; 3 Department of Medical Pharmacology and Physiology, University of Missouri, Columbia, MO 65212, USA

**Keywords:** liver, mitochondrial dysfunction, FAO, MASLD, thyroid hormone, thyroid hormone receptor

## Abstract

Metabolic dysfunction-associated steatotic liver disease (MASLD), formerly termed nonalcoholic fatty liver disease (NAFLD), is a widespread global health concern that affects around 25% of the global population. Its influence is expanding, and it is anticipated to overtake alcohol as the leading cause of liver failure and liver-related death worldwide. Unfortunately, there are no approved therapies for MASLD; as such, national and international regulatory health agencies undertook strategies and action plans designed to expedite the development of drugs for treatment of MASLD. A sedentary lifestyle and an unhealthy diet intake are important risk factors. Western countries have a greater estimated prevalence of MASLD partly due to lifestyle habits. Mitochondrial dysfunction is strongly linked to the development of MASLD. Further, it has been speculated that mitophagy, a type of mitochondrial quality control, may be impaired in MASLD. Thyroid hormone (TH) coordinates signals from the nuclear and mitochondrial genomes to control mitochondrial biogenesis and function in hepatocytes. Mitochondria are known TH targets, and preclinical and clinical studies suggest that TH, thyroid receptor β (TR-β) analogs, and synthetic analogs specific to the liver could be of therapeutic benefit in treating MASLD. In this review, we highlight how mitochondrial dysfunction contributes to development of MASLD, and how understanding the role of TH in improving mitochondrial function paved the way for innovative drug development programs of TH-based therapies targeting MASLD.

## 1. Introduction

Metabolic dysfunction-associated steatotic liver disease (MASLD) is the new term replacing nonalcoholic fatty liver disease (NAFLD). It is the most prevalent chronic liver disease in the Western world, paralleling the steady rise in the prevalence of obesity and its associated metabolic disorders such as hyperlipidemia and type 2 diabetes [1]. Epidemiological studies reported that MASLD affects ~25% of the world’s population [2,3]. In many patients with MASLD, the accumulation of toxic amounts of lipids is accompanied by liver injury caused by oxidative stress characterized histologically by hepatocyte injury (ballooning) and foci of inflammation in the surrounding liver tissue. Histologically, the combination of steatosis, in addition to hepatocyte ballooning and/or inflammation, is called metabolic dysfunction-associated steatohepatitis (MASH), previously termed nonalcoholic steatohepatitis (NASH) [4]. Fibrosis is an important endpoint in MASLD/MASH. Progression to cirrhosis and development of hepatocellular cancer (HCC) are the most feared liver-related complications of MASH [5]. In fact, MASH is projected to be the leading indication for liver transplantation, surpassing other competing etiologies [6]. Further, MASH is the fastest growing cause of HCC in the United States [7]. There are several pathophysiological mechanisms that have been described and implicated in the pathogenesis and progression of MASH. Insulin resistance (IR) is an established risk factor for the development and progression of MASLD. Studies have shown that IR due to chronic nutritional overload renders hepatocytes more susceptible to oxidative stress and mitochondrial dysfunction, which can enhance inflammation and promote severe liver damage [8,9].

Published reports from our group and others document that hepatic mitochondrial dysfunction plays a key role in the progression of MASLD [10,11,12,13,14]. Mitochondrial dysfunction is characterized by various levels of structural damage within the mitochondria, ATP depletion, increased permeability in both the outer and inner mitochondrial membranes and reduced respiratory chain activity, excess production of reactive oxygen species (ROS), and the consequent deleterious deletions in mitochondrial DNA (mtDNA) due to oxidative stress [15]. The metabolic changes seen in MASLD are commonly linked to mitochondrial dysfunction in the hepatocytes. The mitochondrial quality control (MQC) system intricately controls mitophagy, proteostasis, biogenesis, dynamics, and other processes that are essential for maintaining cellular homeostasis. Mitochondrial dysfunction due to failure of MQC is one of the known factors known to perpetuate MASLD [16,17].

Thyroid hormone (TH) has an important role in many physiological processes, including homeostasis, mineral, lipid, carbohydrate, and protein metabolism. TH has an effect on nearly all organs in the body, with the liver representing one of the most important sites of TH action [18]. Thyroid hormones regulate a variety of cell types in the liver along with hepatocytes. The other cell types in the liver include Kupffer cells, stellate cells, and endothelial cells, which have TH receptors and are affected by TH action. While hepatocytes are important players in the liver’s functions and are considerably affected by TH, other liver cell types are also impacted and play a role in mediating TH effects inside the liver [19]. Low TH function can result in hypercholesterolemia, which is believed to be an important step in the pathogenesis of hypothyroidism induced MASLD [20]. TH regulates mitochondrial biogenesis and function in hepatocytes by synchronized nuclear and mitochondrial genome signals [21]. As previously stated, TH performs critical functions in energy and metabolic balance; therefore, it plays a role in the pathophysiology of MASLD. MASLD is closely related to hypothyroidism [22]. TH may play a role in the etiology of MASLD based on studies that suggest disturbances in cellular TH signaling cause MASLD [18,23,24]. This review focuses on the role of TH in mitochondrial dysfunction and potential therapeutic implications of TH in MASLD.

## 2. Mitochondrial Dysfunction and MASLD

Mitochondria account for up to 18% of a hepatocyte’s total volume and carry out vital functions in the hepatic metabolic processes to generate energy [25]. In addition to producing ATP and β-oxidation, mitochondria also generate reactive oxygen species (ROS) and control calcium signaling. Studies have repeatedly shown that impaired mitochondria contribute to the development and progression of MASLD. These derangements include decreased β-oxidation, electron transport chain (ETC) defects, decreased ATP levels, increased ROS generation, cellular damage caused by oxidative stress, and structural alterations in mitochondria [26,27,28]. The alternations in mitochondrial structure and function accentuate the accumulation of lipids in the liver, triggering inflammation and fibrogenesis, and thus contribute to the progression of MASLD [9,26]. During the early stages of MASLD, mitochondria appear to adjust to increased substrate consumption in both humans and mice by boosting β-oxidation, mitochondrial respiration, and ketogenesis [29,30]. However, as MASH progresses, this adaptive response fades, resulting in mitochondrial dysfunction characterized by inefficient β-oxidation, impaired ketogenesis, decreased ATP generation, and electron transport chain leakage [31]. Recent research has established a link between mitochondrial dysfunction and both cell apoptosis and the activation of the inflammasome [32].

### 2.1. ROS and MASLD

Mitochondrial dysfunction results in decreased oxidative phosphorylation and excess ROS production. Increased ROS can cause oxidative stress by oxidizing proteins and peroxidizing mitochondrial membranes, resulting in mitochondrial dysfunction via reduced respiratory chain activity leading to mitochondrial DNA (mtDNA) damage [33]. Furthermore, excessive ROS production has been shown to increase the influx of cytochrome C and other proapoptotic substances through the mitochondrial permeability transition (MPT) channels, resulting in hepatocyte death, a hallmark feature of MASH progression [34,35]. Moreover, previous studies suggest that ROS and lipid peroxidation may enhance TGF-β synthesis in Kupffer cells, activating hepatic stellate cells and increasing the development of collagen-producing myofibroblasts. Collectively, such pathological changes have been shown to promote hepatic fibrosis and, in extreme situations, liver cirrhosis [36]. Further, it has been postulated that ultrastructural abnormalities in mitochondria and an imbalance in mitochondrial dynamics are associated with more severe MASLD/MASH. Failure to eliminate damaged mitochondria can result in an accumulation of defective mitochondria; as such, the liver’s ability to restore normal mitochondrial function deteriorates over time, leading to hepatocyte demise and further progression of MASH [37].

### 2.2. Impaired Mitochondrial Quality Control (MQC) and MASLD

MQC involves several processes such as fission, fusion, biogenesis, and mitophagy. When exposed to oxidative stress, mitochondria use mechanisms such as antioxidants, DNA repair, protein folding, and degradation to maintain their function. Mitochondrial biogenesis, fusion, and fission all serve to compensate for mitochondrial dysfunction in normal physiological and pathological states [38,39]. In the event of cellular injury, mitochondria can be repaired by fusing with healthy counterparts, whereas severely damaged mitochondria undergo fission and are eventually degraded by mitophagy [40,41]. The onset of MASLD is greatly impacted by mitochondrial dysfunction caused by MQC failure [11]. Mitophagy, or mitochondrial autophagy, is an important preventative mechanism against the development and progression of MASLD, as it is responsible for eliminating damaged mitochondria that are highly concentrated inside the cytosol. Mitophagy disorders, on the other hand, have been reported in both MASLD mice models and patients with MASLD. Studies have shown that defects in PINK1 or Parkin cause defective mitochondrial engulfment, thereby worsening MASLD [42]. The inactivation of mitofusin 2 (Mfn2) activity caused by inflammation can have additional deleterious effects on mitophagy, thereby reducing the production of autophagosomes, increasing hepatic steatosis, and accelerating the progression of MASH [43]. Mitophagy appears to be important for both prevention and rescue of MASLD. Recent study suggests that PARKIN-mediated mitophagy may both prevent or slow MASLD progression [44]. Another recent study suggests that upregulation of BNIP-3, which is regulated by SIRT-3 via the ERK-CREB signaling pathway, may be involved in the initiation of mitophagy [45]. Reintroducing Sirt3 restored mitophagy by activating Bnip3 expression, increasing mitochondrial and hepatocyte resistance to lipotoxicity. Mitophagy activity is restored by ERK-CREB signaling, which also maintains mitochondrial homeostasis and reduces hepatocyte apoptosis [45]. Collectively, these data underscore that mitochondrial activity and antioxidant levels in the liver are critical in the pathogenesis of MASLD. A recent study has reported that defective mitochondria is associated with greater risk of MASLD development in those with obesity. In this study, increased formation of ROS in the liver and decreased MQC were shown to be associated with significant decrease in β-oxidation in ~ 50% of patients with MASH [14]. Mitophagy is influenced by hormonal factors. TH has been shown to reduce the severity of MASLD by increasing FAO and stimulating mitophagy and mitochondrial biogenesis [46,47]. TH has also been shown to increase the expression of BNIP3, NIX, ULK1, p62, and LC3 mRNA expression [48]. Mitophagy and mitochondrial biogenesis work synergistically to fine-tune the mitochondrial homeostasis, enabling cells to modify their mitochondrial composition in accordance with cellular metabolic status, stress, and various signals originating from the intracellular environment and hormones.

## 3. Hypothyroidism and MASLD

Hypothyroidism affects 0.2% to 5.3% of the US and European population. It is characterized by an increase in thyroid-stimulating hormone (TSH) levels and a reduction in thyroxine and triiodothyronine hormones. Clinical or overt hypothyroidism occurs when these alterations emerge as hypothyroidism-related symptoms. Subclinical hypothyroidism occurs when TSH levels are increased but TH levels remain normal [49]. It is well-established that hypothyroidism is associated with hypometabolism, commonly manifested as an increase in body weight, decrease in basal metabolic rate, gluconeogenesis, and lipolysis. TH impairment can result in metabolic disorders such as obesity, low lipid metabolism, and insulin resistance, which are commonly associated with MASLD [50]. Both clinical and subclinical hypothyroidism have been linked to MASLD [51]. Several factors contribute to the progression of MASLD in subclinical hypothyroidism. Studies in murine models have shown that TSH affects lipid metabolism via TSH receptors on hepatocytes [52,53,54]. TSH produces hepatosteatosis via the sterol regulatory element binding protein, SREBP [52]. TSH also inhibits hepatic bile acid synthesis via an SREBP2-hepatocyte nuclear factor 4 (HNF)-CYP7A1 signaling pathway [53]. Furthermore, TSH suppresses cholesterol production via increasing AMPK-mediated phosphorylation of hydroxymethylglutaryl-CoA reductase (HMGCR) [54]. These findings document TSH’s independent role in the regulation of hepatic lipid and cholesterol homeostasis. In addition, reduced TH levels cause decreased glucose-sensing receptors in pancreatic β cells, which results in decreased insulin secretion. This results in increased lipolysis in adipose tissue with increased hepatic FFA influx [55,56,57].

Hyperlipidemia in hypothyroidism has been thought to be due to inadequate lipid metabolism due to a decreased number of low-density lipoprotein (LDL) receptors on hepatic cells and an increase in intestinal cholesterol absorption [58]. Increased total and LDL cholesterol levels are thus seen in hypothyroid individuals. MASLD caused by hypothyroidism may occur as a result of increased triglyceride accumulation in the hepatic tissue [56,59]. Lipid accumulation causes oxidative stress and inflammatory reactions within the liver [51]. Leptin has also been implicated in thyroid–liver complex interactions. Leptin levels are elevated in hypothyroid individuals as well as in MASLD patients [24]. Leptin increases insulin resistance in the liver and can contribute to fibrogenesis [24]. Due to mitochondrial dysfunction, individuals with hypothyroidism are also more vulnerable to increased oxidative stress [60,61]. Hypothyroidism is a significant risk factor for MASLD and it has been shown to be associated with impaired glucose and insulin metabolism [62]. Hypothyroid patients have elevated oxidative stress markers; thus, oxidative stress could be the source of hepatocellular damage by decreasing FAO and increasing lipid peroxidation [55,63]. Collectively, these data suggest that TH therapy could be of benefit in hypothyroidism induced MASLD, and therapeutic application of TH (or its analogs) in the treatment of MASLD/MASH offers tremendous promise.

## 4. Thyroid Hormone and MASLD: From Underlying Mechanisms to Therapeutic Implications

TH regulates multiple metabolic activities within cells linked to the metabolism and breakdown of macromolecules, such as carbohydrates, proteins, lipids, and damaged cellular organelles, to maintain homeostasis in various conditions [64,65]. TH plays an important role in hepatic lipid metabolism [66]. According to published reports, individuals with obesity are more likely to have hypothyroidism than those with a normal BMI [67,68,69]. These findings strengthen the hypothesis that TH therapy might be of therapeutic benefit in patients with MASLD with or without hypothyroidism [56].

### 4.1. Mechanisms of Action of TH

The thyroid hormone receptor (TR), a nuclear receptor, controls T3 activity by acting as a T3-inducible transcription factor. TR has two main isoforms: TRα and TRβ, and its expression varies by tissue. TRα receptor is commonly found in the heart, brain, and bone, whereas TRβ is predominantly found in the liver and kidney. TR interacts with thyroid hormone response elements (TREs) in target gene regulatory domains as a heterodimer with another nuclear receptor, the retinoid X receptor (RXR). TR’s recruitment of coregulator proteins regulates target gene expression. The TR/RXR heterodimer binds the nuclear receptor corepressor and silencing mediator of retinoid and TR to inhibit gene transcription via histone deacetylation in the absence of T3. Coactivators are enrolled when T3 is present, whereas corepressors are dismissed, and TH-responsive gene expression occurs [70]. TH promotes de novo lipogenesis in the liver by recruiting the transcription factor ChREBP to the promoters of lipogenic genes via the T3 receptor TRβ1. The lipogenic genes involved are Acetyl-CoA carboxylase alpha (ACACA), fatty acid synthase (FASN), and thyroid hormone-responsive (THRSP).

### 4.2. TH and Its Isoform

THs are synthesized and secreted by the thyroid gland and are required for the control of numerous metabolic processes. The thyroid gland utilizes thyroid follicles as fundamental structures to concentrate iodide and produce the primary THs, namely, 3,3′,5,5′-tetraiodo-L-thyronine (T4) and 3,5,3′-triiodo-L-thyronine (T3) [71]. The anterior pituitary’s thyrotrophs, which release thyroid-stimulating hormone (TSH), regulate TH (mainly T4) secretion from the thyroid gland. By synthesizing and releasing iodinated THs into the bloodstream, the thyroid controls numerous physiological processes in the liver, adipose tissue, central nervous system, cardiovascular system, and musculoskeletal system [72]. Iodothyronine deiodinases (DIO1, DIO2, and DIO3) in extrathyroidal tissue regulate T4 to T3 conversion. Both DIO1 and DIO2 convert circulating T4 to the bioactive TH form, i.e., T3. DIO3 reduces intracellular thyroid by converting T4 and T3 to reverse T3 (rT3) and T2 [73]. Recent studies suggest that T2 has tissue-specific TH activity [18,74].

**Figure 1:**
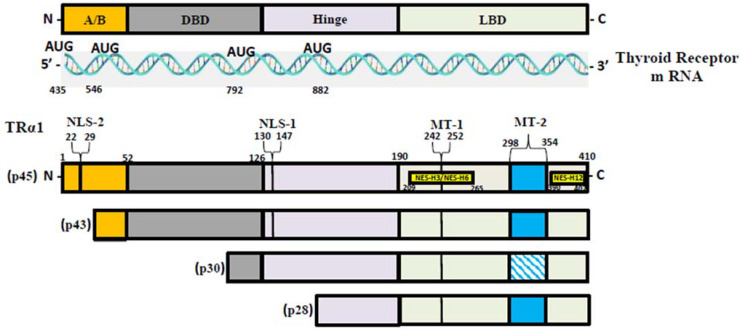
**TR-α isoforms**. Nuclear localization signals (NLS), nuclear export signals (NES), and mitochondrial targeting signals (MT) are shown in TRα1, TRβ1, and TRβ2. Localization signals are positioned in reference to the individual TR domains: N-terminal A/B domain (A/B); DNA-binding domain (DBE); Ligand-binding domain (LBD).

T3, the most active form of TH, binds to two nuclear hormone receptor isoforms (TRα and TRβ). These receptors function as ligand-inducible transcription factors that interact with TH response elements (TREs) found in target gene promoters, enhancers, and intronic regions [64,75]. TRα is the predominant isoform found in the heart, brain, and bone, whereas TRβ is present dominantly in the liver, accounting for more than 90% of TRs in this tissue [65]. TR subtypes are encoded by two genes. The first gene, TRα, is linked to the c-erbA gene on chromosome 17 [76]. The transcription of C-erbA produces three mRNAs: one full-length TRα1, and the other two variants that code for proteins which do not bind with TH [77,78]. The second gene, THRβ, is located on chromosome 3 and shares DNA with c-erbA. TRα1 mRNA contains numerous alternative start sites, resulting in the translation of three shorter isoforms (p43, p30, and p28) based on their kilo Dalton sizes (Figure 1) [76,79,80]. The mitochondrion is a primary site of TH accumulation within the cell [81,82,83]. TR1α p43 is directed to the mitochondrial matrix, whereas TR1α p28 is particularly directed to the mitochondrial inner membrane [81,84,85]. Over the past decacde, it has become clear that T3 not only exerts its effects through a genomic mechanism but also through nongenomic mechanisms mediated by the mitochondrial thyroid receptor isoforms [86]. The nongenomic effects of TH on mitochondrial FAO are the least studied (and least understood) among the metabolic effects of thyroid hormones. In a collaborative study, our group conducted in vitro studies and discovered that the T3-induced increase in mitochondrial FAO is, at least in part, mediated by increased levels of MTP due to increased stability and decreased turnover of the MTP complex [87]. Our data also suggest that the mitochondrial shortened thyroid receptor isoform p43 (mTR) in particular plays an important role in mediating the T3-induced increase in MTP stability [87].

### 4.3. TH and MASLD

TH has been linked to the pathogenesis of MASLD; reduced TH levels have been frequently reported in MASLD patients [88]. According to animal studies, moderate hypothyroidism has been shown to be associated with a higher risk of MASLD [89]. Translational studies of liver transcriptomes from individuals with MASLD undergoing bariatric surgery revealed a decrease in the expression of genes involved in RNA metabolism, protein catabolism, and energy metabolism. These genes, which are controlled by THs under normal physiological conditions, have been shown to have lower expression levels in individuals with MASLD [90]. In rats and humans, lower intrahepatic TH levels in MASLD has been reported [88,91]. TH not only increases de novo lipogenesis (DNL) and improves hepatic insulin sensitivity; it also decreases hepatic gluconeogenesis in hepatocytes. Furthermore, TH promotes lipid export and oxidation [92,93]. The regulation of lipid and glucose metabolism is carried out by the TH receptors, which have direct and indirect effects by interacting with other nuclear receptors such as the peroxisome proliferator-activated receptor (PPAR), liver X receptor (LXR), and bile acid signaling pathways [92]. Several observational studies reported a link between increased serum TSH levels and the presence and severity of MASLD [23,94]. In recently published studies, the regulation of hepatic autophagy and mitochondrial metabolism by TH have been described as crucial steps in hepatic triglyceride metabolism [95,96].

#### 4.3.1. TH and FAO

**Figure 2:**
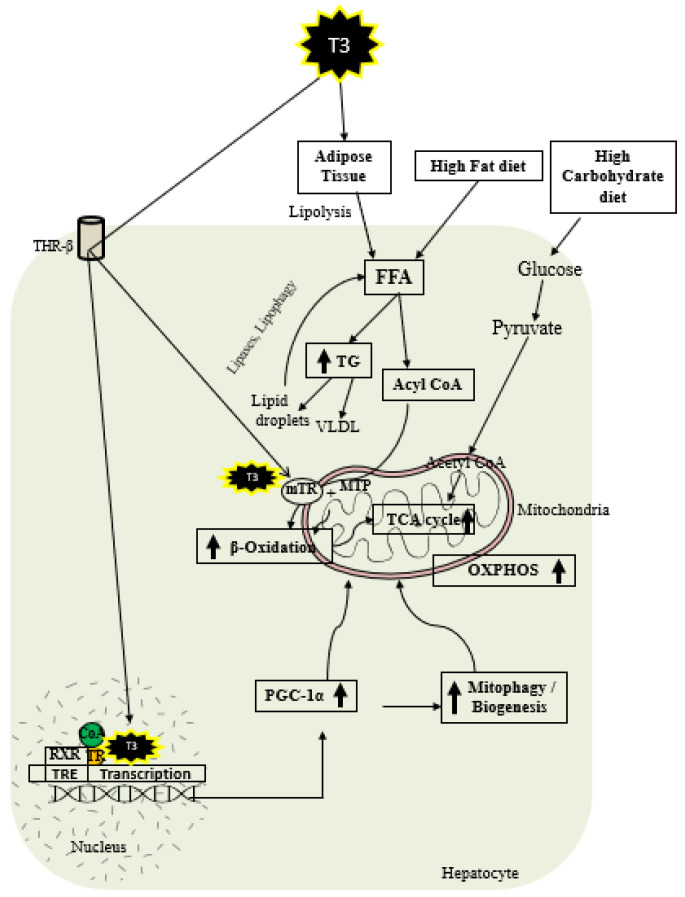
Schematic representations of TH metabolic effects in hepatocytes.

As shown in Figure 2, thyroid hormone increases FAO oxidation, TCA cycle, and oxidative phosphorylation (OXPHOS) via genomic and nongenomic mechanisms. Thyroid hormone increases expression of PGC1 alpha [97,98], which is a master regulator of mitochondrial function. In addition, there is evidence that TH isoform 43 interacts with MTP and improves its activity by improving its stability [87]. TH has been shown to increase fatty acid import, FAO, and oxygen uptake in isolated mitochondria [99]. Previous work suggests that T2 treatment increased FAO when palmitoyl-CoA was utilized as a substrate rather than palmitoyl-carnitine, suggesting that carnitine-palmitoyltransferase 1 (CPT1) could be a potential T2 target, which was further verified by assessing CPT activity [100]. In isolated mitochondria, TH treatment increased the activity of mitochondrial thioesterase, an enzyme responsible for converting acyl-CoA to fatty acid and CoA [86]. We have recently shown that low-dose T3 treatment in mice increased FAO in both chow- and western-diet-fed-animals [101]. The ETC and the tricarboxylic acid (TCA) cycle also play key roles in FAO [96,102], and CPT1 levels are increased indirectly by TH via surtuin 1 (SIRT1) and PPARα [92,103]. TH further increases the amounts of other mitochondrial enzymes required for FAO such as medium-chain acyl-CoA dehydrogenase (MCAD), pyruvate dehydrogenase kinase, and mitochondrial uncoupling protein 2 (UCP2) [104,105,106]. T2 treatment has been shown to promote hepatic FAO in liver mitochondria, increase downstream respiratory activity, increase proton leak, and reduce oxidative stress in the liver mitochondria without causing thyrotoxicity [107,108].

#### 4.3.2. TH and Mitochondrial Biogenesis

TH exerts multiple actions at a molecular level aimed at increasing the number of mitochondria. As shown in Figure 2, T3 promotes mitophagy and mitochondrial biogenesis via peroxisome proliferator-activated receptor gamma coactivator 1-alpha (PGC1α) [48,109]. TH stimulates mitochondrial biogenesis by inducing PGC1α gene expression, which stimulates the transcription of nuclear respiratory factor 1 (NRF1) and mitochondrial transcription factor A (mtTFA) [21]. SIRT1 is activated by TH, which deacetylates PGC1α and increases its ability to bind the regulatory areas of mitochondrial synthesis and function genes [103]. Further, T3 also increases the expression and activation of Unc-51-like autophagy activating kinase 1 (ULK1), which improves the dynamin-related protein 1 (DRP1)-mediated mitochondrial fission, activation, and association of FUN14 domain-containing 1 (FUNDC1) with LC3B, and p62 translocation to mitochondria in hepatic cells [109,110]. T3-mediated mitophagy activation is required for mitochondrial oxidative phosphorylation system (OXPHOS) stimulation [109]. Additionally, T3 promotes mitochondrial biogenesis [110] and increases the rates of mitophagy and mitochondrial synthesis, both of which contribute to enhanced mitochondrial activity and fatty acid β-oxidation.

#### 4.3.3. TH and Mitophagy

To minimize cellular harm caused by reactive oxygen species (ROS), TH induces protective autophagy of mitochondria, a process called mitophagy [109]. Mitophagy is initiated by excessive ROS production from mitochondria, leading to the release of intracellular Ca^2+^, activation of calcium/calmodulin-dependent protein kinase 2 (CAMMK2), phosphorylation of AMP-activated protein kinase (AMPK), and subsequent activation and translocation of ULK1 to the mitochondria after AMPK phosphorylation [109]. Elevated ROS levels are perpetuators of mitophagy, which ensures the preservation of mitochondrial quality required for β-oxidation of fatty acids and oxidative phosphorylation. Mitophagy induced by ROS generation selectively sequesters damaged mitochondria to be removed from the cell, preventing additional oxidative damage and cell death [109]. Recent research suggests that PGC-1α is involved in the complex regulation of mitochondrial quality beyond biogenesis, including mitochondrial network dynamics and autophagic removal of damaged mitochondria [111].

### 4.4. Potential Therapeutic Use of TH and Its Analogs in MASLD

In recent years, THs, as well as the TR-β agonists and additional liver-specific analogs, have been studied as a potential MASLD treatment [23]. Research from previous studies reported the use of T3 to promote weight loss in patients with obesity and to treat hypercholesterolemia [112]. T3 injections given daily intraperitoneally (ip) to ob/ob mice have been found to decrease both body weight and fat while increasing oxygen intake and oxidative metabolism [113]. GC-1, a novel TR-β agonist, has been reported to reduce the development of hepatic steatosis and lipid peroxidation in mice [46] and decrease hepatic TG levels with no major side effects [114,115]. MB07811, another orally administered TR-β agonist, has been shown to prevent hepatic steatosis in rats and mice via boosting β-oxidation and mitochondrial respiration rates, lowering hepatic TG levels and stimulating CPT1α expression [47]. Resmetirom (MGL-3196) has been shown to be effective in lowering hepatic TG, lipid peroxidation, ALT, steatosis, inflammation, and fibrosis in animal models [116,117]. Increased mitochondrial β-oxidation has been suggested to be one of the mechanisms by which Resmetirom decreases liver fat [118]. VK2809 therapy has been shown to reduce hepatic lipid accumulation in a glycogen storage disease Ia (GSD1a) mouse model by restoring the autophagy, mitochondrial biogenesis, and β-oxidation of fatty acids [119]. KB2115, a TR-β agonist, has been reported to decrease the total and low-density lipoprotein (LDL) cholesterol levels in the blood and to prevent the development of hepatic steatosis [120]. Ongoing research in our laboratory has demonstrated that low-dose T3 is effective in increasing hepatic mitochondrial FAO and reversing MASLD in mice [121]. Based on these results, our group recently initiated a randomized double-blinded placebo-controlled clinical trial to test whether low-dose thyroxine (T4) is effective in improving the histological features in Veterans with biopsy proven MASH (National Library of Medicine NCT05526144) [122]. Taken all together, the results of the above studies demonstrate that TH therapy might be an effective strategy in treating MASLD/MASH. The use of TH and its analogs in preclinical and clinical research is summarized in Table 1.

## 5. Conclusions

MASLD poses a considerable public health problem with major socioeconomic impact. The onset and progression of MASLD is a multifactorial process influenced by genetic, epigenetic, and environmental factors. The development of MASLD is directly linked to mitochondrial dysfunction, as mitochondria play an important role in the β-oxidation of FFAs and are the principle intracellular generators of ROS. Recent studies focusing on understanding the role of TH in hepatic lipid metabolism and defective autophagy, mitophagy, and mitochondrial function in health and disease have shed light on the role of TH and mitochondrial dysfunction in the development and progression of MASLD. TH therapy, TRβ1 analogs, and liver-specific synthetic analogs in preclinical models and preliminary studies in patients with MASLD have shown promise as safe and potentially useful strategies in the treatment of MASLD.

## Figures and Tables

**Table 1:** Effects of TH and its analogues in MASLD.

Compound	Model and Dose	Study Findings	MASLD Impact	References
Animal Studies				
**TH**				
T_2_	Hepatocyte isolated from Wistar rats; 10^−7^ to 10^−5^ M	Reduction of acyl-CoA oxidase and peroxisomal β-oxidation	Reduction of hepatic lipid accumulation	[123]
T_2_	C57BL/6J mice; 2.5 µg/100 g; ip	Increased fatty acid oxidation and decreased lipogenesis	Inhibition of fat accumulation in liver	[124]
T_2_	Male wistar rats; 25 µg/100 g; ip	Reduced hepatic fatty accumulation, enhanced fatty acid oxidation rate and carnitine palmitoyl transferase activity	Activates mitochondrial processes, reverses hepatic steatosis	[125]
T_2_	Rats injected with 25 µg/100 g; ip	Reduction in Serum TG and cholesterol	Prevents fatty liver by increasing fatty oxidation	[107]
T_3_	ob/ob mice; 25 µg/100 g; ip	Lowered body weight and fat, increased oxidative metabolism	Increased oxidative metabolism in brown adipose tissue and liver	[113]
T_3_	Male wistar rats; 25 µg/100 g; ip	Promotes fatty acid peroxisomal and mitochondrial β-oxidation	Prevents hepatic fat accumulation by increasing β-oxidation	[46]
T_2_ and T_3_	Wistar rats; 25 and 2.5 µg/100 g; ip	Increased CPT-1 levels	Lowering hepatic lipid content, induced autophagy and intra-hepatic acylcarnitine flux	[126]
T_4_	Male C57BI/6J mice;	Decreased hepatic triglyceride and cholesterol	Reduce hepatosteatosis and prevent MASH progression	[127]
**Thyroid hormome analogues**				
T_3_ and TRβ agonist GC-1	Male fischer rats; 4 and 5 mg/kg; ip	Marked fatty liver with mild hepatitis	Prevents fat accumulation by increasing mitochondrial and peroxisomal oxidation, complete regression of liver steatosis	[46]
TRβ agonist GC-1	Male sprague Dawley rats; 1 µg/kg; oral gavage	Reduction in hepatic TG levels	Treatment of obesity and hypercholesterolemia	[114]
MB07811	Male sprague Dawley rats, ob/ob mice; 1 to 50 mg/kg; oral gavage	Prevents hepatic steatosis, reduced plasma FFA and triglycerides	Increased hepatic fatty acid β-oxidation and mitochondrial respiration rates, as well as lower hepatic triglyceride levels and stimulation of CPT1α expression	[47]
Resmetirom (MGL-3196)	C57BI/6J mice; 3 mg/kg for 8 weeks by oral gavage	Lower hepatic triglycerides, lipid peroxidation, steatosis, inflammation and fibrosis	Improvement in systemic and hepatic metabolism	[116]
VK2809	GSDIa mouse model; 10 mg/kg; Subcutaneously	Restoring autophagy, mitochondrial biogenesis, and β-oxidation of fatty acids	Reduced hepatic lipid accumulation	[119]
GC-1 and KB-2115	Male Sprague-Dawley rats; 164 and 100 µg/kg; ip	Increased white adipose tissue lipolysis	Reduced hepatic steatosis	[128]
TG68	C57BL mice; 2.8 mg/kg in drinking water	Reduction in liver weight, hepatic steatosis and triglycerides	Can be used in MASLD	[129]
TRC150094	Male wistar rats for 8 weeks; ip injection (0.750 mg/100 g b wgt	Reduction of Fat accumulation	Can be used in MASLD	[130]
**Clinical Trials**				
**TH**				
MGL-3196 (Resmetirom)	36 weeks randomized trial in patients with biopsy proven MASH with fibrosis given 80 mg orally daily	Significant reduction of hepatic fat, liver enzymes, lipoprotein, inflammation and fibrosis.	Patients showed reduction of hepatic fat compared to placebo, adverse events were mild and moderate	[131]
	2 weeks randomized trial with 0.25 to 200 mg/day	Significant reduction of total cholesterol and triglycerides	Safe and showed beneficial effect on lipid parameters	[132]
KB2115 (eprotirome)	5-day randomized trial in patients given 50 to 2000 µg orally daily	Reduction in serum TC and LDL in overweight patients	Reduced body weight	[120]
VK2809	12-week study of low dose of 5 mg in patients	Reduction in LDL levels	Improvements in liver fat content in patients with MASLD	[18]
Levothyroxine (T_4_)	Patients with type 2 diabetes and steatosis given 18.75 µg/day	Low dose T_4_ decreased lipid content in euthyroid male patients with type 2 diabetes mellitus.	Safety and efficacy of TH therapy for MASLD in men	[91]
DITPA	8-week randomized trial in patients with dose from 90 till 360 mg/d	Lowered serum cholesterol and decrease in triglycerides	Reduced body weight	[133]

## Data Availability

Not applicable.
